# Awareness of Constipation and Its Complications Among the General Population of Saudi Arabia

**DOI:** 10.7759/cureus.53030

**Published:** 2024-01-27

**Authors:** Lujain Alshareef, Khalid H Alnafei, Ibrahim S Alibrahim, Abdullah M Alsharif, Teyf M Althubiani, Hadeel T Mandurah, Rani Alsairafi

**Affiliations:** 1 College of Medicine and Surgery, Umm Al-Qura University, Makkah, SAU; 2 Department of Obstetrics and Gynecology, Maternity and Children Hospital, Makkah, SAU; 3 College of Medicine and Surgery, Department of General Surgery, Umm Al-Qura University, Makkah, SAU

**Keywords:** cross-sectional study, general population, cross sectional study, saudi arabia, complications, constipation

## Abstract

Background: Constipation is a common gastrointestinal issue with significant economic implications. Its prevalence varies globally, and it can have acute or chronic forms with primary or secondary causes. The pathophysiology of constipation is influenced by various factors. While previous studies have reported varying levels of awareness of constipation and its complications in different regions of Saudi Arabia, no comprehensive research has assessed such awareness among the general population. This study, thus, aimed to assess awareness of constipation and its complications among the public in Saudi Arabia.

Methodology: A descriptive cross-sectional study was conducted in Saudi Arabia on individuals aged 18 and above, excluding healthcare workers. Data were collected using a validated survey distributed online between September 2023 and November 2023.

Results: A total of 1,139 participants were included in the study, predominantly female 739 (64.9%), and most of the participants 595 (52.2%) aged between 19 and 30 years, The majority of participants 850 (74.6%) had bachelor's degree. Regarding the source of medical information, 312 (27.4%) of the participants stated that they obtained medical information from social media. Additionally, 811 (71.2%) of participants reported experiencing constipation. Overall, 847 (74.4%) of participants demonstrated a good level of awareness of constipation, while 292 (25.6%) had a poor level of awareness. Significant associations were found between awareness level and age, city of residence, and occupation.

Conclusion: Most adults in Saudi Arabia have a high level of awareness of constipation and its complications.

## Introduction

Constipation is described by the American College of Gastroenterology as unpleasant defecation marked by difficulty passing stools, infrequent stools, or both [1]. Constipation is a widespread gastrointestinal issue that has significant monetary costs for communities [2]. Its prevalence is thought to range from 1% to 80% globally [2]. Constipation can be classified based on its duration as either acute or chronic and can be further categorized based on its underlying causes as either primary or secondary [3,4]. The pathophysiology of the disease is influenced by a variety of variables, including types of diet, hereditary factors, intestinal motility, absorption, socio-economic level, daily activities, biology, and drug use [5]. Constipation, particularly chronic constipation, is linked to a number of issues, most of which arise in situations ‏that the sufferer ignores [6,7]. The most commonly reported related problems are hemorrhoids, anal fissures, fecal impactions, rectal prolapses, and the development of inguinal hernias [6,7]. Adults without known or suspected secondary causes of constipation are diagnosed using the Rome IV diagnostic criteria [8]. An anorectal function test is often used in advanced examinations to assess defecatory problems [9,10]. The difference between slow transit constipation and regular transit constipation is determined by colonic transit investigations [9,10].

A cross-sectional study to evaluate the general population’s awareness of constipation in the western region of Saudi Arabia reported that 70% of its 778 participants were well aware of constipation and its complications [11]. Their level of knowledge was influenced by their age, academic position, and personal or family history of constipation [11]. A different study conducted in Riyadh found that 75% of its 1,855 participants were fully aware of constipation and its implications [12] and revealed a notable variance in awareness between age groups [12]. Moreover, a positive connection has been established between married status and awareness [12]. According to a recent study in the central region of Saudi Arabia, only 4.4% of the population suffered from constipation, with 95.6% of the selected participants stating that they did not experience constipation [10]. However, females (79.2%) were more likely than males (20.8%) to experience constipation [10]. Additionally, constipation tends to be more severe in individuals between the ages of 20 and 35, while it disappears entirely in people over the age of 51 [10]. Furthermore, the findings indicate that constipation more frequently affects non-smokers, people who take fiber-rich foods only once a week, people who are under chronic stress, and people who are dehydrated [10]. As far as we know, no study has been done to determine the public’s degree of awareness of constipation throughout all regions of Saudi Arabia. Therefore, our study aimed to assess awareness of constipation and its possible complications among the general population of Saudi Arabia.

## Materials and methods

Study design and setting

A comprehensive cross-sectional study was conducted on individuals from the general population aged 18 years and above in Saudi Arabia. The study was carried out between September 2023 and November 2023. To collect the required data, a survey that had been validated beforehand was distributed online to the participants.

Ethical considerations

This research study was approved by the Institutional Research Board (IRB) at Umm Al-Qura University in Makkah, Saudi Arabia, with approval number HAPO-02-K-012-2023-09-1743. The survey responses were obtained without any personal identification of the participants, and their confidentiality was strictly maintained using a system of codes, numbers, and pseudonyms. To ensure a secure and successful study, participants were given complete information about the study’s research objectives, data collection methods, and safety measures. Before commencing the study, participants were required to sign a consent form.

Eligibility criteria

Our research focused on all individuals of either gender aged 18 years and above who lived in Saudi Arabia and expressed an interest in participation. Only those who worked in healthcare and those who refused to participate were excluded.

Sample size

OpenEpi version 3 (www.openepi.com) was used to determine a sample size of 384 based on the general population of Saudi Arabia [13]. Nonetheless, we were able to reach 1,232 people who agreed to participate in our study. After eliminating healthcare workers from the sample, we obtained a total of 1,139 valid responses for our research.

Data collection and management

A validated questionnaire was developed for this study based on an extensive literature review regarding constipation awareness among the public [11]. The data were collected through an online questionnaire distributed by data collectors using Google Forms. To ensure that the questionnaire could be easily understood by the target population, it was translated into Arabic. The questionnaire comprises two sections: the first section collected demographic data and the second section assessed the degree of awareness of constipation and its possible complications.

Statistical analysis

A scoring system was implemented to assess the participants’ knowledge about constipation. Each correct answer was awarded one point, while an incorrect response received a score of zero. Participants were classified based on the number of correct answers they provided. Those who correctly answered 60% or more of the questions were considered to have a high level of constipation awareness.

For analysis, the data were collected, reviewed, and entered into Statistical Package for Social Sciences version 26 (SPSS; IBM SPSS Statistics for Windows, Armonk, NY). After translating the data from Arabic to English and coding the variables, it was found that the data were statistically significant at a p-value of ≤ 0.05. Descriptive analysis was conducted via percentages and prescribing frequency distributions for the study variables, including participants’ data. The chi-square test was used to assess the significant associations between sociodemographic factors and constipation awareness levels.

## Results

A total of 1232 agreed to participate in this study after excluding the healthcare workers and those aged less than 18 years old. The total number of participants is 1,139. Specifically, 739 (64.9%) participants were females, while 400 (35.1%) were males. Regarding participants’ age, most of the participants (595, 52.2%) were aged from 19 to 30, while 240 (21.1%) were aged from 31 to 40. Moreover, 176 (15.5%) of participants were aged from 41 to 50, while those aged from 51 to 60 were 94 (8.3%), and only 34 (3.0%) were aged above 60 years. Further analysis shows that 358 (31.4%) of participants were from the western region, while 246 (21.6%) were from the eastern region. Moreover, participants from the southern region were 188 (16.5%), while 172 (15.1%) of the total participants were from the northern region. Additionally, 157 (15.4%) of participants were from the central region. Regarding participant education, the data show that most of the participants' education level was bachelor's degree (850, 74.6%), while 256 (22.5%) had high school education. Furthermore, regarding the source of medical information, 312 (27.4%) of participants state that they take medical information from social media, 309 (27.1%) they take medical information from healthcare workers, while 283 (24.8%) take medical information from family and friends, and only 235 (20.6%) of participants they depend on online articles as a source of medical information. Further details about socio-demographics are demonstrated in Table 1. 

**Table 1:** Socio-demographic characteristics of the participants and their associations with the awareness level

Sociodemographic factors		n (%)	Level of awareness		P-value
			Poor level of awareness,n(%)	Good level of awareness, n(%)	
Gender	Female	739 (64.9)	183 (24.8)	556 (75.2)	0.394
	Male	400 (35.1)	109 (27.2)	291 (72.8)	
Age	19-30	595 (52.2)	193 (32.4)	402 (67.6)	0.001
	31-40	240 (21.1)	36 (15.0)	204 (85.0)	
	41-50	176 (15.5)	31 (17.6)	145 (82.4)	
	51-60	94 (8.3)	25 (26.6)	69 (73.4)	
	>60	34 (3.0)	7 (20.6)	27 (79.4)	
City of residence	Western region	358 (31.4)	106 (29.6)	252 (70.4)	0.012
	Eastern region	246 (21.6)	73 (29.7)	173 (70.3)	
	Southern region	188 (16.5)	36 (19.1)	152 (80.9)	
	Northern region	172 (15.1)	43 (25.0)	129 (75.0)	
	Central region	175 (15.4)	34 (19.4)	141 (80.6)	
Level of education	Elementary school	16 (1.4)	7 (43.8)	9 (56.3)	0.248
	Intermediate school	17 (1.5)	5 (29.4)	12 (70.6)	
	High school	256 (22.5)	71 (27.7)	185 (72.3)	
	Bachelor	850 (74.6)	209 (24.6)	641 (75.4)	
Source of medical information	Family and friends	283 (24.8)	82 (29.0)	201 (71.0)	0.060
	Social media	312 (27.4)	89 (28.5)	233 (71.5)	
	Online articles	235 (20.6)	47 (20.0)	188 (80.0)	
	Health care workers	309 (27.1)	74 (23.9)	235 (76.1)	
Monthly income	Less than 1000 riyals	362 (31.8)	106 (29.3)	256 (70.7)	0.164
	From 1000 to 5000 riyals	305 (26.8)	80 (26.2)	225 (73.8)	
	From 6000 to 10000 riyals	204 (17.9)	45 (22.1)	159 (77.9)	
	More than 10000 riyals	268 (23.5)	61 (22.8)	207 (77.2)	
Occupation	Employee	507 (44.5)	150 (29.6)	357 (70.4)	0.011
	Retired	90 (7.9)	25 (27.8)	65 (72.2)	
	Unemployed	542 (47.6)	117 (21.6)	425 (78.4)	

**Figure 1:**
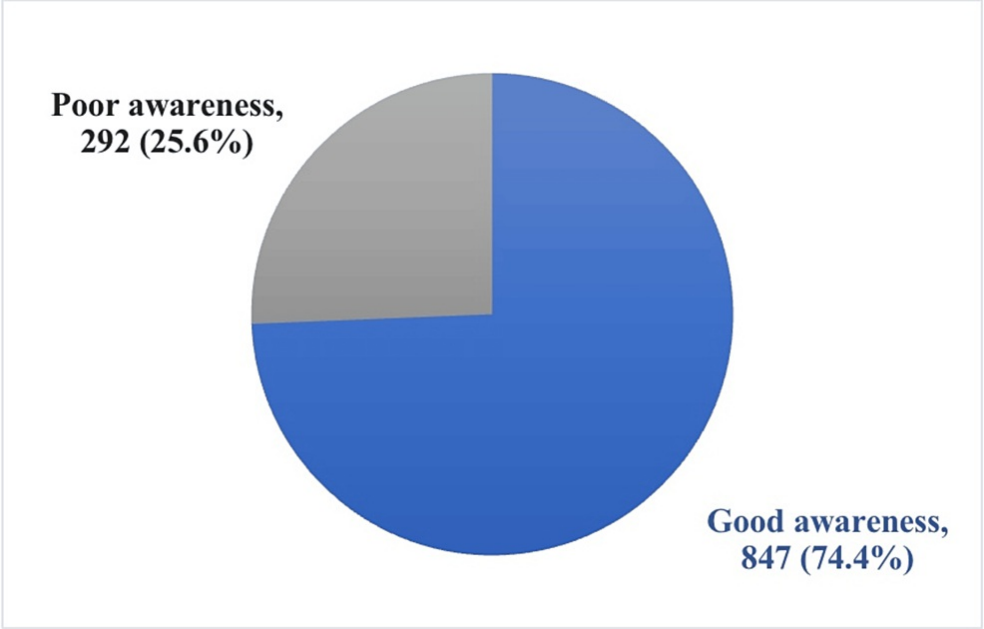
Participants’ awareness about constipation

Table 2 displays the participants’ answers to the awareness questions about constipation. Moreover, 811 (71.2%) of the total participants stated they currently have constipation or have had constipation in the past. The majority of the participants believe that constipation will affect a person’s quality of life (1,009, 88.6%). Participants were identified as having a good or poor level of awareness toward constipation based on their correct answers. Most of the participants (847, 74.4%) had a good awareness level, while 292 (25.6%) had a poor awareness level toward constipation (Figure 1).

**Table 2:** Participants' awareness about constipation and its complications

Items	Agree, n(%)	Disagree, n(%)	I don’t know, n(%)
1- Constipation means that a person has three or fewer bowel movements in a week.	628 (55.1)	136 (11.9)	375 (32.9)
2- Constipation is a common condition in Saudi Arabia.	796 (69.9)	67 (5.9)	276 (24.2)
3- Lack of exercise is a risk factor in the development of constipation.	874 (76.7)	106 (9.3)	159 (14.0)
4- Advanced age increases the likelihood of developing problems associated with bowel movements.	885 (77.7)	80 (7.0)	174 (15.3)
5- Inadequate dietary fiber is a predisposing factor in the development of constipation.	1003 (88.1)	39 (3.4)	97 (8.5)
6- Constipation could be due to Idiopathic/unknown causes.	570 (50.0	235 (20.6)	334 (29.3)
7- Causes of constipation vary from simple dietary causes to severe malignancy causes.	882 (77.4)	52 (4.6)	205 (18.0)
8- Constipation is a major risk factor for developing anorectal conditions like (haemorrhoids, hernias, and anal fissures).	998 (87.6)	34 (3.0)	107 (9.4)
9- Very painful abdominal pain associated with complete constipation requires emergent medical/surgical care.	715 (62.8)	136 (11.9)	288 (25.3)
10- Adequate water intake is critical in preventing the development of constipation.	1027 (90.2)	46 (4.0)	66 (5.8)
11- Regular visits to primary care is crucial in the management of constipation and its related complications.	861 (75.6)	80 (7.0)	198 (17.4)
12- The use of over-the-counter constipation treatment is effective in the treatment of constipation.	427 (37.5)	450 (39.5)	262 (23.0)
13- The cost of treatment of constipation influences the decision to seek medical services.	339 (29.8)	311 (27.3)	489 (42.9)
14- Do you currently feel you have constipation or have had constipation in the past?	811 (71.2)	225 (19.8)	103 (9.0)
15- Do you think constipation will affect a person’s quality of life?	1009 (88.6)	53 (4.7)	77 (6.8)

The association between the awareness level regarding constipation and the socio-demographic factors was assessed and shown in Table 1. There was a statistically significant association between the awareness level with age (0.001), city of residence (0.012), and occupation (0.011). There was no statistically significant association between the awareness level with gender (0.394), level of education (0.248), source of medical information (0.060), or monthly income (0.164).

## Discussion

This study aimed to assess the level of knowledge among the Saudi population about constipation and its possible complications. Constipation is a common gastrointestinal problem that can lead to various medical conditions that affect the quality of life [14]. Therefore, early detection and proper treatment are necessary to prevent such complications [15]. This study found that 74.4% of participants had a high degree of awareness about constipation and its possible complications, while the rest had a low level of awareness. A previous study carried out in the western portion of Saudi Arabia had similar results, with 70% of the sample demonstrating an adequate level of understanding regarding constipation [11]. Furthermore, a survey conducted in Riyadh revealed that 75% of the public had a sufficient level of understanding of constipation [12]. Although our results in general considered primarily those with a sufficient level of awareness of constipation, it was also ascertained that only 50% of our population agreed that constipation may occur due to unexplained causes. In comparison, the western region study found slightly higher results (59%), while the Riyadh survey revealed 54% agreement [11,12].

Chronic constipation causes can be classified as primary (idiopathic) or secondary causes including metabolic, due to medication, and neurological disorders [16,17]. Despite that, both primary and secondary causes shared similar treatment. However, secondary sometimes requires additional therapy [18]. This finding highlights the importance of health educational programs to raise public understanding of constipation's causes and associated treatment options.

Regarding risk factors, our study shows that 23.3% of participants either disagreed with or did not know that lack of exercise is a main contributor to constipation. Findings from studies done in the western area (29.5%) and Riyadh (28.3%) show slightly higher findings [11,12]. Moreover, 88.1% of the participants in our study agreed that a diet containing insufficient fiber can lead to constipation. A similar study conducted in the western region, in which 84% of the sample participated, found that 88.5% of participants in Riyadh agreed with the statement [11,12]. Moreover, this study revealed a positive correlation between the level of understanding and participant age. Similar to other studies in the literature, older participants possessed a greater understanding of constipation than younger participants [11,12].

Additionally, we discovered a positive correlation between constipation awareness and the city of residence in Saudi Arabia. Compared to other regions, individuals from the central region exhibited higher levels of understanding. This is due to the fact that Riyadh, the capital of Saudi Arabia, has the largest number of medical facilities and primary healthcare centers in the country, and these aid residents in raising their level of awareness [19]. This finding also highlights the importance of implementing health education programs about constipation and its possible complications in other parts of Saudi Arabia.

Limitations

This study, evaluating public awareness of constipation across all regions of Saudi Arabia, has some limitations. Although an e-mail posted on the first page of the survey was intended to answer participants’ questions, some misunderstandings may have resulted due to the online self-reported nature of the survey. Moreover, we used a convenience sampling method, which may have affected the generalizability of the study.

## Conclusions

This study aimed to evaluate the degree of awareness about constipation and its possible complications among the citizens of Saudi Arabia. We found a positive correlation between the awareness level about constipation and its possible complications and several factors, including city of residence, occupation, and age. Therefore, we recommend emphasizing health education sessions to increase awareness, particularly among those groups with the lowest level of understanding. Moreover, as we observed that social media was the primary platform for accessing medical information, we suggest utilizing social media as an effective tool for disseminating information and raising awareness about constipation and its possible complications.

## References

[1] Brandt LJ, Prather CM, Quigley EM, Schiller LR, Schoenfeld P, Talley NJ (2005). Systematic review on the management of chronic constipation in North America. Am J Gastroenterol.

[2] Sanchez MI, Bercik P (2011). Epidemiology and burden of chronic constipation. Can J Gastroenterol.

[3] Herz MJ, Kahan E, Zalevski S, Aframian R, Kuznitz D, Reichman S (1996). Constipation: a different entity for patients and doctors. Fam Pract.

[4] Rao SS, Rattanakovit K, Patcharatrakul T (2016). Diagnosis and management of chronic constipation in adults. Nat Rev Gastroenterol Hepatol.

[5] Andromanakos N, Skandalakis P, Troupis T, Filippou D (2006). Constipation of anorectal outlet obstruction: pathophysiology, evaluation and management. J Gastroenterol Hepatol.

[6] Nickerson AJ, Rottgen TS, Rajendran VM (2021). Activation of KCNQ (KV7) K+ channels in enteric neurons inhibits epithelial Cl− secretion in mouse distal colon. Am J Physiol Cell Physiol.

[7] Alessi CA, Henderson CT (1988). Constipation and fecal impaction in the long-term care patient. Clin Geriatr Med.

[8] Jamshed N, Lee ZE, Olden KW (2011). Diagnostic approach to chronic constipation in adults. Am Fam Physician.

[9] Bharucha AE, Dorn SD, Lembo A, Pressman A (2013). American Gastroenterological Association medical position statement on constipation. Gastroenterology.

[10] Alhassan M, Alhassan A, Alfarhood A, Alotaibi K, Alrashidy N, Alshalhoub K, Almeshal M (2019). Prevalence of constipation among central region population, Riyadh and Qassim provinces, Saudi Arabia, 2018-2019. J Family Med Prim Care.

[11] Hemdi M, Alkarmo MY, Alahmadi RA, Almajnoni RS, Alharbi JK, Alfahmi AM, Almaghrabi HA (2023). Awareness of the general population toward constipation and its complications in the Western Region, Saudi Arabia. Cureus.

[12] Ahmed S, Alshahrani A, Alhazzaa A, Alslaihem M, Benragosh N, Alswayah M (2020). Awareness of adult population toward constipation and its complications in Riyadh, Saudi Arabia. IJMDC.

[13] Sullivan KM, Dean A, Soe MM (2009). OpenEpi: A web-based epidemiologic and statistical calculator for public health. Public Health Rep.

[14] Diaz S, Bittar K, Hashmi MF, Mendez MD (2023). Constipation. StatPearls.

[15] Forootan M, Bagheri N, Darvishi M (2018). Chronic constipation: a review of literature. Medicine (Baltimore).

[16] Passos MD, Alvariz RC, André EA (2022). Diagnosis and management of chronic idiopathic constipation: a narrative review from a Brazilian expert task force. Arq Gastroenterol.

[17] Bharucha AE, Lacy BE (2020). Mechanisms, evaluation, and management of chronic constipation. Gastroenterology.

[18] Basilisco G, Coletta M (2013). Chronic constipation: a critical review. Dig Liver Dis.

[19] Al-Sheddi A, Kamel S, Almeshal AS, Assiri AM (2023). Distribution of primary healthcare centers between 2017 and 2021 across Saudi Arabia. Cureus.

